# Surgery in the New College

**Published:** 1882-12-20

**Authors:** 


### *SURGERY IN THE NEW COLLEGE.*

A number of interesting and important surgical operations have been performed in the Clinics of the Southern Medical College during the present term of Lectures. Photographs of one of these, a very large tumor, involving the parotid gland, has been shown to us with the promise that the same will be reported with illustrations.

